# Cerebellar liponeurocytoma, a rare tumor: Case report and review of the literature

**DOI:** 10.1016/j.ijscr.2021.105937

**Published:** 2021-04-30

**Authors:** Yousef S. Abuzneid, Hussam I.A. Alzeerelhouseini, Sundus Shkokani, Wafa Aqel, Asad Aldarawish

**Affiliations:** a Al-Quds University Faculty of Medicine, Jerusalem, Palestine; b Makassed Islamic Charitable Society, Jerusalem, Palestine

**Keywords:** Liponeurocytoma, Cerebellum, Posterior fossa, Tumor, Case report

## Abstract

**Introduction:**

Cerebellar liponeurocytoma is a rare tumor of the central nervous system occurring mainly in the posterior fossa, which shows neuronal and variable astrocytic differentiation, along with foci of lipomatous differentiation.

**Case presentation:**

Herein, we describe a 50-year-old female patient who presented to the hospital complaining of headache, tinnitus, and vertigo with positive cerebellar signs. MRI revealed a left cerebellar tumor. After tumor resection, histological examination and immunohistochemistry were done and the diagnosis of cerebellar liponeurocytoma was confirmed.

**Discussion:**

Liponeurocytoma may be mistaken as a medulloblastoma with lipidized cells or a lipomatous ependymoma. Histopathological examination, reinforced by immunohistochemistry and electron microscopy, are required to distinguish between these entities. The rarity of this tumor and paucity of pertinent information regarding its biological potential and natural history have resulted in the application of various treatment modalities.

**Conclusion:**

Liponeurocytoma is a rare benign tumor with cerebellum is the typical site for it. Although surgery is the treatment of choice; however, postoperative radiotherapy may have a role in case of incomplete tumor resection or recurrence.

## Highlights

• Cerebellar liponeurocytoma is a rare benign tumor that mainly occurs in the posterior fossa.
• Surgery is the first line in management.
• Postoperative radiotherapy has a role in case of incomplete tumor resection or recurrence.

## 1 Introduction

Liponeurocytomas are rare adult cerebellar tumors that are characterized by well-differentiated histological features, low proliferative activity, and a favorable prognosis. According to the new WHO classification in brain tumors, it is recognized as a separate clinicopathological entity, distinct from medulloblastoma [[1], [2], [3]].

The prognosis for these patients in the long-term is unknown, however, some papers suggest a benign clinical course, with a small number of late tumor recurrences after surgery [2]. Patel et al. revealed that 42 cases of cerebellar liponeurocytoma were reported in the English literature between 1978 and 2009, with a male to female ratio of 22:20 [4].

Immunohistochemical staining reveals neuronal as well as glial differentiation. Homer Wright rosettes and pseudorosettes may be seen too [5].

The clinical symptomatology of this condition includes symptoms of cerebellar dysfunction, such as gait disturbance and uncoordinated movements, which are slowly progressive and generally not detected in the early stages of the disease. Vomiting and progressive visual symptoms may appear too as a result of increased cranial pressure in the later stages of the disease [6].

Preclinical diagnosis is difficult as to date, no typical imaging features have been identified, however, Aker et al. reported that the lesion exhibited heterogeneous enhancement on T1-weighted images [7].

Although findings from computed tomography (CT) and magnetic resonance imaging (MRI), and pathological and immunohistochemical characteristics have been reported in around 70 cases, clinical and pathological diagnosis of liponeurocytoma remained challenging and the characteristics of this tumor entity are still poorly understood [8].

The work has been reported in line with the SCARE criteria [9].

## 2 Case presentation

A 50-year-old female patient, known case of hypertension (HTN) and taking *normatin* as a medication, was in her usual state of health until 6 months prior to admission when she started to complain from headache, tinnitus and vertigo. She sought medical advice and was treated as otitis media (according to the patient).

Before ten days from her admission to our hospital, she had episodes of imbalance while she was walking and recurrent vomiting.

**Fig. 1:**
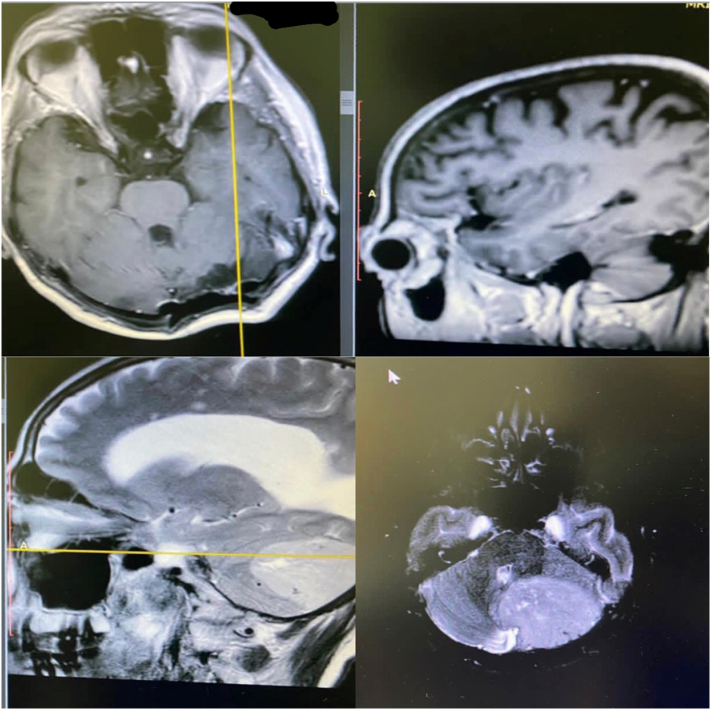
Brain MRI showing a left cerebellar mass of unknown origin which was further analyzed pathologically giving us a diagnosis of liponeurocytoma.

Once again, she sought medical help and, after a brain CT and a brain MRI were done, the doctors found a left cerebellar tumor (Fig. 1). In the upcoming days, she was referred to our hospital in April 2019 for further evaluation and surgical management.

The patient came to our hospital by her own (using public transportation) because there were no facilities for the tumor management in other places and there was no need to being transported by ambulance.

We did a physical examination focused on a neurological examination due to her symptoms and diagnosis. The patient was conscious, oriented and alert, with a Glasgow coma scale (GCS) of 15/15.

The cranial nerves were intact, and she was positive for cerebellar signs. She also was positive for the Romberg sign, had an ataxic gait and could not perform the tandem gait.

The patient then underwent surgery in which a midline suboccipital craniectomy with microscopic gross total resection (GTR) of the left cerebellar tumor and a cranioplasty under neuromonitoring was performed successfully.

The surgery was smooth and without intraoperative complications with an estimated blood loss of 500 cc.

After the surgery, the patient was transferred to the ICU in good conditions with stable vital signs, conscious, oriented, alert and a GCS of 15/15. The cranial nerves were intact, she was able to move all limbs freely and the wound was dry and clean. Afterwards, she was discharged in good conditions, with stable vital signs and no complications or sequela.

According to the histology department, the specimen that they received in formalin consisted of a multiple pieces and fragments of a grayish tissue measuring in aggregate 7.5 × 5 cm grossly. Further microscopic investigations of the histologic sections showed a biphasic tumor with a mixture of neurocytes and lipidized cells in which the neurocytes were arranged in density cellular sheets of monotonous cells with scanty and often clear cytoplasm, rounded to oval nuclei and salt-and-pepper chromatin. Lipidized cells were clustered and resembling mature adipocytes.

Rare mitotic figures were noted and were investigated. The tumor cells were positive for synaptophysin and neuron specific enolase (ESN) immunostains, focal for glial fibrillary acidic protein (GFAP) and negative for epithelial membrane antigen (EMA).

With all these histological findings, it was concluded that the sample was consistent with a cerebellar liponeurocytoma, WHO grade 2.

After a month from the operation, the patient came to the out-patient clinic for follow-up in which we could see that she was in good conditions, with no complains and the cerebellar signs were gone. Since there was no residual tumor left after the surgery and there were no symptoms showing us cerebellar involvement, there was no need for our patient to take radiotherapy once she was discharged.

Since the patient did not come to us again, we suspect that she is not complaining from any other symptom in the neurosurgical field.

## 3 Discussion

A liponeurocytoma is a neurocytic neoplasm that involves a variable extent of neuronal differentiation in addition to a focal lipomatous and astrocytic differentiation that can be seen due to a consistent immunoreactivity to synaptophysin, neuron-specific enolase (NSE) and MAP-2. In addition, the tumor cells may be positive for nuclear NeuN but negative for cytoplasmic IDH1- R132H, epithelial membrane antigen (EMA), cytokeratin (CK), and nuclear Olig-2 [8,10].

This kind of disease also shows a monotonous pattern of isomorphic round to oval cells with focal accumulations of lipid-laden cells, that, at the same time, they seem to be neuroepithelial tumor cells with lipid accumulation or they constitute a form of lipidization instead of true adipose cells [11,12].

In the lipomatous cells, the lipid vacuole membranes were positive for SYN, MAP-2, vimentin and S-100 and the expression of GFAP and S-100 is limited to scattered reactive astrocytes [1,8,13].

Within a radiological exam, liponeurocytomas show a more or less well-demarcated hypointense mass with attenuation values of fatty tissue on CT scans. It may be associated with parenchymal cysts or cerebellar hemorrhage. However, MRI of a cerebellar liponeurocytoma normally shows an isointensity on T1 weighted imaging, a heterogeneous intensity on T2 weighted imaging, and a high intensity on diffusion weighted imaging. Edema can be also observed in the surrounding [14].

Usually, the symptoms that are present with this condition are headache, vomiting, nausea and dizziness, as well as ataxia with unsteadiness and frequent falls. A flow obstruction of the cerebrospinal fluid can also be seen [[11], [12], [13]].

There is no clue about the genetic and cellular origins of this kind of tumor and it is a rare condition that is usually found in the cerebellum, although supratentorial lesions have been described and seem to share common immunohistological profiles and neoplastic behavior [15].

With all this symptomatology, there is some differential diagnosis that we should think about and they include oligodendrogliomas, clear cell ependymomas and high-grade tumors like medulloblastomas. It is easy to misdiagnose a liponeurocytoma with these other differential diagnoses, but we can differentiate them [8,15,16].

Oligodendrogliomas can show similar pathological findings demonstrating neuronal differentiation with glial and neuronal characteristics; however, oligodendrogliomas show highly positive results of Olig2 immunohistochemistry in contrast to liponeurocytomas.

In addition, a chromosome 1p/19q co-deletion is shown in 50–80% of oligodendrogliomas, while 1p/19q deletion is not observed in liponeurocytomas and also oligodendrogliomas show a high frequency of isocitrate dehydrogenase 1 (IDH1 R132H) mutation in addition to 1p/19q co-deletion [16].

Regarding the differentiation between a medulloblastoma with lipidized cells and lipomatous ependymomas, they can be differentiated using immunohistochemical panels. Negative reaction for EMA rules out the possibility of an ependymoma and older age of presentation (43.66 ± 15.60 years) and extensive lipomatous change combined with a lower Ki-67/MIB-1 proliferation index favors a diagnosis of a liponeurocytoma [8].

The primary treatment of choice for a liponeurocytoma is surgery but there are some controversial thoughts for the post-operative treatment strategy that is still in discussion.

This kind of tumor has a recurrence rate of 20 to 32% in a mean time of 8 to 10 years after surgery and it is more common in patients where total resection could not be achieved.

For this reason, additional radiotherapy has to be discussed in those patients [8,16].

As an important information to be mentioned, 50% of the patients who did not receive an adjuvant therapy suffered from a tumor recurrence. That is why adjuvant therapy seems to have a favorable impact on tumor recurrence as it is discussed in many papers [8].

As mentioned above, our patient suffered from headache, recurrent vomiting episodes and ataxia with unsteadiness in addition to tinnitus and vertigo that, at the beginning, a doctor attributed to an otitis media and treated her according to that with no improvement since it was a wrong diagnose. After that, the doctors were able to find a cerebellar tumor by doing a CT scan and MRI, so it was referred to us for a further surgery that was successful and in which the doctor was able to resect the tumor totally, so no radiotherapy was needed.

## 4 Conclusion

Liponeurocytomas are rare benign tumors occurring mainly in the cerebellum. It should be differentiated from other types of posterior fossa tumors due to the drastic difference in the treatment plan with histopathological examination and immunohistochemistry, being the investigations of choice.

Surgical resection is the mainstay for management, and adjuvant radiotherapy post-operation seems to be sufficient in those cases with incomplete tumor resection or recurrence.

However, additional studies are necessary to determine clinical features and the exact prognosis.

## Ethical approval

The study is exempt from ethical approval in our institution.

## Sources of funding

This research did not receive any specific grant from funding agencies in the public, commercial, or not-for-profit sectors.

## CRediT authorship contribution statement

Study concept or design: Asad Aldarawish.

Data collection and data analysis: Yousef S. Abuzneid.

Writing the manuscript: Yousef S. Abuzneid, Hussam I. A. Alzeerelhouseini, Sundus Shkokani, Wafa Aqel.

Writing the manuscript: Yousef S. Abuzneid, Hussam I. A. Alzeerelhouseini, Sundus Shkokani, Wafa Aqel.

## Guarantor

Dr. Yousef S. Abuzneid.

## Research registration number

Not applicable.

## Consent

Written informed consent was obtained from the patient itself for publication of this case report and accompanying images. A copy of the written consent is available for review by the Editor-in-Chief of this journal on request.

## Declaration of competing interest

There is no conflict of interest.
